# Food Acquirability: An Unexplored Component of Food Security?

**DOI:** 10.3390/foods13132052

**Published:** 2024-06-27

**Authors:** Emeka Franklin Okpala, Lilian Korir, Louise Manning

**Affiliations:** 1 School of Agriculture, Food and Environment, Royal Agricultural University, Stroud Road Cirencester, Gloucestershire GL7 6JS, UK; emeka.okpala@student.rau.ac.uk; 2 Lincoln Institute for Agri-Food Technology, University of Lincoln, Riseholme Park, Lincoln LN2 2LG, UK; lkorir@lincoln.ac.uk

**Keywords:** food security, household, decision maker, food insecurity, access to resources, time poverty, acquirability

## Abstract

The key elements, or pillars, of food security are stated as food availability, food access, food utilization, and stability. These food security pillars are often linked to food security interventions at the national, community or household level. However, if the urban ‘household’ is the unit of interest for any food security intervention, this research asks if a more holistic element, or pillar, is needed. The aim of this research has been to explore the socio-economic aspects of food security/insecurity that we have termed as a result of the research “food acquirability”. Through the use of structured questionnaires (n = 120), and analysis of the data derived from local market and supermarket settings in the city of Awka, Nigeria, the concept of food acquirability has emerged and been conceptualized and critiqued. The contribution of this paper is to frame the concept of acquirability with regard to food security in Nigeria in order to develop a better understanding of the factors that impact household urban food security/insecurity and how they can be effectively mitigated. Factors of acquirability that emerged were culture, time poverty, resource availability and cooking skills, and household food preference and meal choice.

## 1. Introduction

Food security occurs when all persons have, at all times, access to sufficient, nutritious and safe food both economically and physically which meets their food choice and dietary needs for a healthy and active life [1]. The four pillars of food security, food availability, food accessibility, food utilization and stability are the essential elements used in policy development to assess a wide range of interrelated social, economic and environmental factors affecting people’s ability to be food secure [2,3,4]. Food availability is the presence of food in a given location. Food accessibility implies the ability to physically access food (influenced by available infrastructure) or financially access food (influenced by available income). Food utilization describes the use of food in terms of the right quality and quantity, and stability refers to the continuous/or uninterrupted access to food, now and in the future. Hence, these four pillars of food security have been used to critique the functionality of a food system, and the interrelationship of social, economic and environmental factors that affect societies and communities in present and future times [2,3,5].

Being food-secure reflects the ability to access, or have ownership of resources, appropriate levels of income and assets to enable personal strategies and contingencies that can ease the impact of shock or squeeze situations in the food system [5,6]. Conversely, food insecurity is said to occur when people lack constant access to safe and nutritious food sufficient for growth and healthy development, and necessary for an active and fulfilling life [7]. Globally, food insecurity is linked with various adverse health outcomes such as diabetes, heart disease, hypertension, and challenges with mental health and physical health functioning [8,9,10]. Food insecurity, depending on its duration, can either be chronic or transitory [11]. Chronic food insecurity is acute, and relates to extreme hunger, starvation and vulnerability conditions [11], and is directly associated with the level of poverty prevalent in a given society [11,12]. Transitory food insecurity is associated with a short-term or temporary situation of hunger which can be influenced by sudden socio-economic shocks, i.e., a reduction in an individual’s ability to access, afford or produce food [13]. Severe food insecurity is synonymous with people who have no food and have lived for days without eating; these individuals are said to be “hungry” [7]. The FAO further notes that food insecurity may be the result of unavailability of food and/or the lack of resources required to acquire or produce food [7]. Factors that influence food insecurity include a reduction in a country’s ability to produce enough food, the lack of infrastructure to assure food security and/or changes to government policies that impact a food system, such as an unpredicted rise in the price of food, household size, household income, and/or busy schedules of working people. [11].

The COVID-19 pandemic has also contributed to household food insecurity, affecting national economies causing inflation and high levels of employee redundancy with children and women experiencing the greatest impact [14]. Over the course of the pandemic, food insecurity conditions were linked to short-term intervention behaviors such as “panic buying/shopping” or “shop looting”, as in the case of South Africa [15]. However, the challenges of food availability, in retail locations or through food production, and food access in the long term were experienced before the pandemic [16]. The ongoing Ukraine/Russia conflict has impacted on food supplies for many countries around the world further exacerbating the problem of food insecurity [17]. The FAO measures food insecurity based on the “Food Insecurity Experience Scale (FIES)” which considers these and other strategies which are an indication of economic difficulties related to challenges in obtaining food [7]. They include the following components:

- Uncertainty about food access. This group of people is food secure but may experience mild food insecurity.
- Compromise on food variety and food quality. This group of people lacks the ability to choose their food and faces moderate food insecurity.
- Skipping meals and reducing the quality of food eaten. This group of people experience moderate food insecurity, and
- No food for a day or more than one day. These are people who face acute food insecurity.

The FAO further highlights that people experiencing moderate food insecurity do not have the quality and quantity of food required to sustain themselves, with a level of uncertainty about their ability to obtain food mainly due to a lack of resources or money to purchase food [7]. Micronutrient deficiency, otherwise known as “hidden hunger” affects over 2 billion people, 1 in 4 of the global population [18,19]. Devastatingly, its effect varies from poor health and weakened immunity to mental impairment, low economic productivity and even death [19]. Additionally, hidden hunger relates to poor intake of micronutrients that are essential for the human body to function. This does not imply that affected individuals do not have sufficient food intake; rather, it places emphasis on the quality of food being consumed [19]. Hidden hunger does not only affect human health; it’s impact on social development, especially in low- and middle-income countries is high [19]. Failure to afford food will increasingly affect a person’s exposure to certain forms of malnutrition, irrespective of the amount of food available nationally or regionally. This can lead to stunting in children in some locations or, alternatively, malnutrition due to the consumption of energy-dense but nutritionally poor foods that can lead to obesity, experienced both by children and adults [20,21,22]. Food insecurity is closely associated with household and national socio-economic factors such as poverty, unemployment, household size, income, levels of food waste, and food prices [5,22,23,24]. However, food insecurity in some situations may extend beyond conditions of poverty, or conditions influenced by poverty, e.g., low assets ownership, and low human capital development in terms of skills, training and education and a lack of access to finance [9]. In summary, food insecurity continues to be a global intractable challenge. Around 820 million people globally, are undernourished [18], and micronutrient deficiencies remain a concern, especially in sub-Saharan Africa. This is despite the continued efforts of governments, non-governmental organizations, and aid organizations that support income interventions, food aid and activities that enhance food production [25]. Whilst the FAO differentiates in terms of levels of food insecurity and with regard to pillars of food security, if the “household” is the unit of interest for any food security intervention, a more holistic approach is required. This approach is described in this paper as considering food acquirability.

The aim of this research has been to iteratively explore socio-economic aspects of food acquirability to inform future empirical research and to consider how this framing may inform more focused food security related policy interventions that operate at the household level. The acquirability of food, at the household level, is driven by uninterrupted physical, social and financial access to food, while minimizing food waste to ensure that sufficient safe and nutritious food is available and affordable for human consumption at all times.

## 2. Literature Review

Food insecurity is understood to impact individuals and/or households at different levels of severity, and households are also often seen to employ different types of coping strategies to manage the food shortage. These coping strategies could include limiting the number of people they can feed, borrowing or begging for food, and rationing food within the household—indicating different degrees of food insecurity, with more severe strategies suggesting a higher severity [26]. Household poverty influences household food security [27]. Though Nigeria is one of the largest and fastest growing economies in Africa with over 236.7 million people, 25.3 million people are said to face acute food insecurity and 40.1% of the population is below the poverty line [28]. In 2019’s data—the timeframe of the data collection in this study— the level of poverty in Nigeria was 40% of the overall population, with approximately 83 million people affected [29]. These data show the challenges faced by Nigeria, especially with availability and affordability of food [30]. The majority of Northern households in Nigeria (84.9%) are food poor with more male respondents (95.45%) who are food poor compared to their female counterparts (4.52%), with a higher proportion in rural areas (83.4%) and over half of respondents (57.5%) having no formal education [27]. The average per-capita expenditure on food within the study area [27] was NGN 25,524.36 USD 128.23) annually or 0.35 USD/person per day. Child poverty is rising in Northern Nigeria, driven by a weak national economy and inflation [31]. Indeed, another study found that level of education affected the food security status of households where teachers oversaw either food purchases or food preparation within the household, i.e., they are making decisions within the household [32]. The study found in two urban locations (Lagos and Ibadan) that three quarters of households were food-insecure and around a third (30.6%) were experiencing hunger. Food insecurity was related to the level of household income, but the educational status of secondary school teachers versus primary school teachers played a role as the findings showed that households of the former were more food secure. A study in Benue State found that 53.3% of rural households and 62.2% of urban households were able to meet the recommended calorie intake of 2500 Kcal per person per day [33]. However, focusing on calories does not necessarily mean the individuals within the households are nutrient-secure. In terms of the factors influencing the food security status of households, the study asserted that there were three factors: age, household size and household income. This means that factors such as level of unemployment, household income, household size, age, educational level of the household head, etc., relate to the inquiry of this study.

Bourlakis and Weightman [34], building on the findings in [35], explained the role of the household food decision maker in terms of six different roles:

- Initiator: The person who recommends what food the family should buy;
- Influencer: The person whose suggestions or views influences the food decision process;
- Decider: The person who makes the final decision on what the household can afford or should buy;
- Buyer: The actualizer of the household decision and purchasing plan;
- User(s): The person(s) who consume or use the commodities/services sourced or purchased;
- Gatekeeper: The person who keeps track of information that relates to the food purchase decision processes.

Household income and family size often determine household food decision making [36]. The role of gender, inequality and cultural bias that lessens/reduces female access to finance, employment, and education can limit their influence on household food decision making especially in Africa [37], i.e., the social construction of what it is to be female, or not, in a given context.

Household roles constitute one of the key influencers in consumer decision making including the location where food is purchased [38], especially the roles of the father and mother within the household or the demographic shift from individual consumer to the family consumption unit when it comes to food choices and purchasing decisions [39]. However, other family members including children can still play a role [28,40]. The roles household members can play can change, i.e., they are dynamic influenced by occupation and income, household size and health status [41]. Gender does play a role in influencing who emerges among spouses within a household as the main food decision maker. This trend has been identified in [36], a study conducted in Kenya, and [39], a study conducted in Anambra State, Nigeria. The findings indicate that the household food decision making process is mainly dominated by males (e.g., husbands or fathers) rather than females. Mothers (n = 567) were participants in another study in Anambra State, Nigeria. Findings revealed that 61% of households had experienced food insecurity in the previous 12 months, and those households where mothers had low levels of education were more likely to be food-insecure [42]. Age, income and education were also shown to have some effect, all of interest in this study. The household food decision maker was selected as the response variable of choice because it underpins gender in the food decision making process and the power dynamics within the household, e.g., who makes the decisions about food purchases within the household, what is the influence of the income level of the household, what is the influence of the educational status of household decision maker, and what is the influence of the size of the household? The next section considers the materials and methods of the study.

## 3. Materials and Methods

The research was undertaken in Anambra State, Nigeria, in the capital city, Awka. Anambra State is situated in the southeast geographical zone in Nigeria. The state has a land area totaling 4416 sq km with 21 local government areas (LGA) with about 177 communities. Anambra State is urbanizing, with over 62% of its population residing in urban areas [43]. Awka lies due west of the Mamu—the river forming part of the Eastern boundary of the State—and the land area of Awka covers a 10km radius and is heavily populated. The pragmatic research paradigm employed in this study focuses on the nature and consequences of human actions in a social context [44,45]. Data were collected between December 2019 and February 2020 from individuals (n = 120); the inclusion criteria meant that respondents must be 18 years of age or older and must voluntarily consent to be involved in the study. The respondents, sixty in each food purchase location, were convenience sampled and interviewed using a structured questionnaire proforma at the Eke Awka market (local market) and a supermarket, pseudonymized as Supermarket A. Descriptive and inferential statistical analyses were undertaken. Multinomial logistic regression (MLR) was used as the data set; this consists of a dependent variable that was categorical in nature with two or more unsequenced or unordered levels [46]. In practical sense, MLR gives rise to multiple possible outcomes as opposed to just one outcome [46]. We used the MLR model in accordance with the following six assumptions: (1) The dependent variable is measured at the nominal level. (2) The data set had one or more independent variables that are either ordinal, continuous or nominal. (3) Observations are independent, and the dependent variable has mutually exclusive and exhaustive categories. (4) Multicollinearity does not exist amongst variables, i.e., this occurs when two or more independent variables have high correlation within the data set. (5) A linear relationship exists between the continuous independent variables and the logistic transformation of the dependent variable involved in the data set. (6) No outliers. By using the MLR, the independent variables can either be continuous, categorical or both [47]. The equation used was

(1) $$Y=\log(\frac{p}{1-p})\;=\;a+b_{1}X_{1}+b_{2}X_{2}+b_{3}X_{3}+b_{4}X_{4}+b_{5}X_{5}+b_{6}X_{6}+e_{i}$$

where Y = household food purchase decision maker (a proxy for food access (one of the pillars of food security)), X_1_ = market choice (food price), X_2_ = market choice (quality of food), X_3_ = age of respondent, X_4_= marital status, X_5_ = challenges faced with food acquisition, X_6_ = occupation, X_7_ = household size, X_8_ = education status, X_9_ = household income, and X_10_ = frequency of market visit.

The rationale for this approach is guided by other research in the area. Ali et al. [48] carried out a study on the determinants of food security in Rangpur city, Bangladesh, using a logistic regression model. The regression analysis indicated a number of variables as important in explaining food security status of households. The number of dependents or household size impacts on food security as household income is spread across the number of individuals in a household. Age was shown to have a positive effect on food security, suggesting that the older the household members the more established their incomes. Education status was shown to have a positive effect on food security, so higher levels of education influenced employment status and household income. Additional variables are assessed in this study. The variable of interest in this study is the household decision maker.

In terms of language barriers as a limitation to data collection, the study location was the home state of the lead author, so this limited potential language barriers. In addition to this, the lead author speaks the English language and the Yoruba languages, as well as his native tongue, the Igbo language. It is for these reasons the study location was selected. The use of convenience sampling means that both representativeness and generalizability of the findings needs to be reflected on, especially where there could be self-reporting bias by the respondents, for example, not being truthful in their declaration of their income or other demographic status.

## 4. Results and Analysis

Across the respondents, those in the age range of 26–45 years were the highest represented in the local market, supermarket and combined data set, where the combined data set includes combined figures from markets, 75%, 70% and 72.5%, respectively (Table 1). This was followed by respondents within the age ranges of 18–25 and 46–65. Female respondents were more highly represented among the study population than men. In the combined data set, 66.7% of respondents were married, while 24.2% were single, 7.5% were widowed, while only one respondent was divorced and another, a widower. In the local market (Eke Awka), respondents with household size 3–6 had the highest representation (45%) within the sample population, then individuals who live on their own, followed by respondents with a household size of 7–10 (one fifth of the data set). Respondents in the supermarket showed a different profile, with a family size of 3–6 being the most represented, followed by single individuals. This implies that the majority of respondents are within the household size of three to ten people (67.5%). More so, single individuals and persons living on their own are represented as the second highest population when the three sets of data are compared. This compares with a household mix of 2–4 people (17.88%) and 5–9 people (40.90%) reported in NBS [29] for Nigeria.

The local market respondents most frequently reported having attained a secondary school leaving certificate (45%), followed by those with a bachelor’s degree certificate (30%). However, in the supermarket setting, respondents were observed to have a bachelor’s degree (43.3%), then a secondary school leaving certificate (36.7%). In the local market, self-employed respondents were most frequently represented (53.3%) within the sample population, followed by those who are fully employed (16.7%), while in the supermarket, respondents who are self-employed were the most frequently recorded (36.7%), followed by fully employed respondents (33.3%), being double that of the respondents from the local market. Interestingly, only three respondents stated they were unemployed compared with an unemployment rate of 34.24% for male and 24.13% for female reported in the national data [29]. This means that the sample population in this study reported a higher employment rate than was captured with the NBS survey, which summarized the unemployment rate in Nigeria as a whole. Similarly, the NBS (Q2) report for unemployment/and underemployment, showed that the unemployment rate in Anambra State at the time of the study was 27.1%, while those who are underemployed was 28.6% [49]. The unemployment rate indicates that more people within Anambra State are either unemployed or underemployed than the proportion shown amongst respondents in this research. The fewer respondents captured could have just been as a result of their presence at that study location at the time of data collection, or respondents may have been too embarrassed to reveal that they were unemployed (i.e., there was a reporting bias), or it could simply just be the case of a skewed sample population compared to the general city population, or even both. This is an important consideration when reflecting on the findings of this study. The data set also suggests that the majority of respondents in the study are self-employed, which implies that they own their urban business which ordinarily exist on a small scale. Examples include being hairdressers, tailors, mechanics, electricians, etc.

Respondents in the survey from the local market (Eke Awka) (Table 1) reported a household income of NGN 18,000–NGN 40,000 per month (n = 23; 38.3%) and NGN 41,000–NGN 80,000 per month (n = 20; 33.3%), with these two groups having the highest frequency within the sample population. Similarly, in the supermarket setting, respondents with household incomes of NGN 41,000– NGN 80,000 per month (n = 18; 30%), and a household income of NGN 18,000– NGN 40,000 per month (n = 15; 25%), were reported, while households within the income range NGN 81,000– NGN 100,000 represented nearly a quarter of the respondent’s population (n = 14; 23.3%). The NBS, in a report about poverty from September 2018 to October 2019, states that 40% of the population (82.9 million people) lived below the poverty line of NGN 137,430 (USD 381.75) a year or NGN 11,450 per month NBS [29]. This figure represents urban and rural populations. This means that the majority of households in the study population can be said to be food-insecure. According to the World Bank Group, a person can be said to be living below the poverty line if food expenditure and consumption is below USD 1.90 per person per day [50]. With regard to the data collected, this study’s “household size” averaged 4.5 people (obtained from the household data of this study). If household size is compared with household income, with the exchange rate USD 1 = NGN 367 (as of 30 March 2020), NGN 80,000 equates to an average household income per month of USD 218 or USD 7.79 per day. With an average household size of 4.5, this means that an average income of less than USD 1.73 per day is available for all expenditure for 77.6% of the sampled population respondents. This statistic, however, assumes that the household income is spent equally across all members of the household. This depth of analysis was not a key determinant of this particular study, but this overview finding is worthy of further analysis in future studies of this nature. The aim of the research has not been to determine which household is in poverty, or relative poverty according to a baseline figure either, but positioning the sample population against such data are of value when interpreting the findings of the study.

Food price was stated as the main driver of consumers’ market choice for 91.7% of the respondents in the local market (Table 2). In the supermarket, food quality was much more influential, as 96.7% of the surveyed population stated this factor primarily influenced choice. Other factors (location, food variety, loyalty, and distance to market) were of less influence, but they still had some effect on choice of food market. The distinguishing feature in the responses between the local market and the supermarket (price and quality of food), showed a difference in terms of being price-related or wanting convenience. While convenience attributes are primarily associated with supermarket food consumers, they may also perceive the quality of the food in the market (local) as not appropriate to their needs.

Giroux et al. [51] concluded that, despite the continuous rise in the number of supermarkets in South Africa, informal food vendors remain an important source of food for households. This trend could be influenced by physical factors, economic factors and/or social factors. With regard to physical factors, food consumers may consider factors associated with infrastructure including: the type of building(s) or road networks that lead to the market, traffic, electricity within the market for food preservation,, etc. The economic factors of influence include food price, quantity of food, food availability, quality for the price they pay (value), etc. While the social factors relate to the general environment (i.e., market infrastructure and market hygiene) and the level of interaction between the seller and the buyer, including the level of bargaining power for consumers [51]. Road traffic was stated by 44 respondents (36.7%) in the combined data set as the main challenge they face when acquiring food; this was followed by time constraints (33.3%). A bad road network was also a factor of influence. Interestingly, time was not an important factor for those respondents at the local market, as only eight people reported this as a major challenge (13.3%); however, this was the most important factor for respondents at the supermarket (53.3%). In the combined data set, that includes data from both markets. High transportation cost (4.2%) and distance to market (2.5%) were both identified as factors of influence but had a low response rate. Only two respondents (1.7%) noted that they faced no challenge whatsoever in acquiring food (Table 3). This implies that road traffic in this urban area (the study location, Awka) is a major challenge faced by respondents in acquiring food and can be attributed to poor infrastructural development within the city.

Respondents who noted that “time” was of major concern stated this was more challenging in responses associated with the supermarket rather than the local market. Hence, food consumers in the supermarket may be prepared to pay higher prices to offset a perceived lack of time. Similarly, this may reflect the busy lifestyle of the Awka working-class and business owners, where fast food entrepreneurs grew quickly in numbers in the state to provide fast, on-the-go meals for the majority of business owners and working-class people of Awka metropolis, who consider themselves time-poor [52]. Perceptions of time poverty were drawn through into the conceptualization of acquirability.

**Figure 1:**
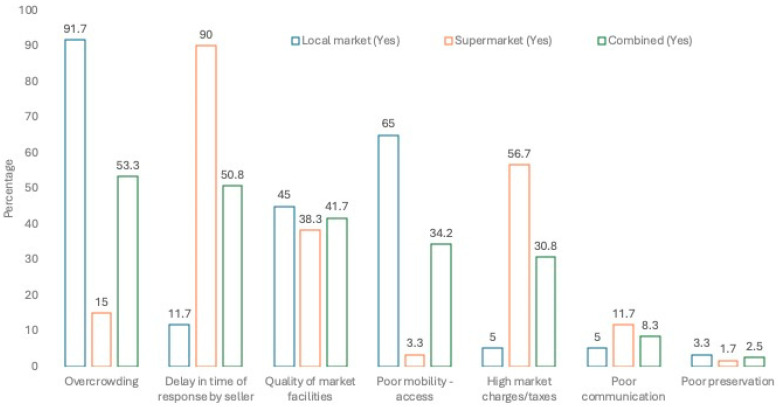
Infrastructural challenges reported by respondents in market locations.

**Figure 2:**
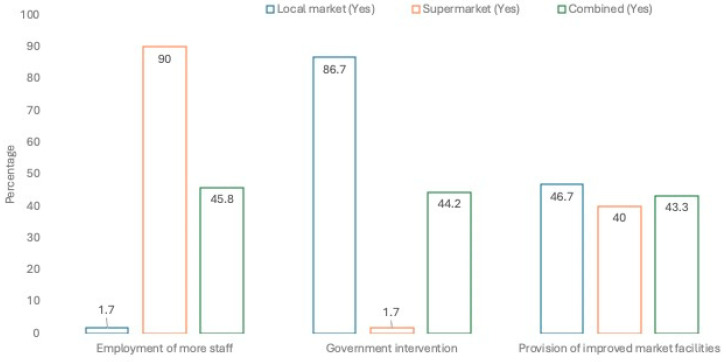
Opportunities for market improvement.

Some infrastructural challenges were specific to either the local market or the supermarket respondents; others are common to both. In the local market, “over-crowding” (n = 55; 91.7%) was significantly more important, while “poor mobility network-footpath access” is slightly more of a concern for those accessing the local market (Figure 1). In the local market, it was clear that infrastructural challenge exists for those seeking to access the market, while, in the supermarket, among the sample group, “delay in time of response by the seller” was stated by 90% of respondents as being the challenge encountered by food consumers. In the combined data set, “high market charges” (n = 61; 50.8%) appeared to be a concern for half of the respondents among the study group who were then asked about options to improve the market channel (Figure 1, where the Yes responses are shown). In the local market, respondents proposed government intervention (n = 52; 86.7%) for improving food market infrastructure for the convenience of both the food sellers and food consumers who visit the market. On the other hand, in the supermarket, the employment of more staff (n = 54; 90%) was found to be more important to respondents as an improvement within its setting (Figure 2, where the Yes responses are shown). Finally, in the combined data set of both the local and supermarkets, no variables were found to be significantly more important than others across both locations mainly because of the skewed response in each group for these two factors and similar responses for improvement of facilities.

**Figure 3:**
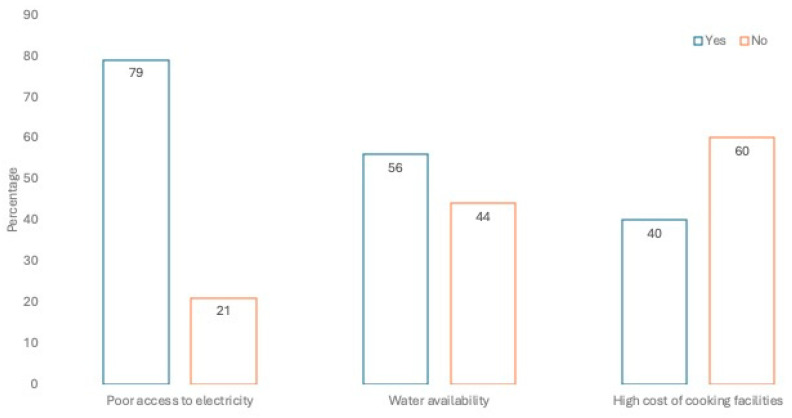
Infrastructural challenges reported by respondents in preparing and storing food.

Just over half (57.5%) of respondents from the combined data set noted that at least 1% of waste is generated from the raw food that they purchased, prepared, and cooked for their consumption. The remainder (42.5%) stated that at least 2–5% of waste is generated from the food they purchase. Three respondents generated 6–15% of waste from their utilization of food. Elsewhere in South Africa, one study found that household food waste emerged mostly from excess food buying, excess food preparation, poor food storage, poor purchase planning and falling for special offers and over purchasing. The higher the household income, the more likely people were to waste food [53]. Respondents in this study appear more judicious with the food they purchase, perhaps attributed to the proportion of low-income earners among the study population. Poor facilities to store food in the home were reported in the Awka study, where poor access to electricity was stated by respondents (79.2%)—see Figure 3. Poor infrastructure, such as living where there is no access to electricity or the electricity supply is intermittent, might limit the respondent’s desire to purchase food in a large quantity or cook in a large quantity.

Smit [54] identified multiple issues that affect food safety, food retail, food governance, urban and peri-urban agriculture in Africa and urban food systems in Africa. Peculiar to Nigeria, the unavailability of potable water and the lack of adequate infrastructure (including electricity) influences the safety of food at all levels (household, street vendors, supermarket, etc.) [54]. While respondents in this study could invest in generator sets, these are expensive to buy and maintain, and impact the environment causing air pollution (Figure 3); however, water availability and the cost of cooking facilities were not seen as significant limiting factors in terms of infrastructure. These findings led to resource availability and cooking skills being a theme in the conceptualization of acquirability.

The regression analysis is summarized in Table 4 and Table 5. Table 4 details the list of variables regressed for the purpose of this study. Fourteen variables (this includes the dependent and independent variables) from a total of n = 120 respondents were included in the regression analysis.

Pseudo R-square

The pseudo-R-square measure include the Cox and Snell (0.728), Nagelkerke (0.843) and McFadden (0.655). The model accounts for 65.5% to 84.3% of the variance and reflects a relatively acceptable sized effect.

Likelihood Ratio Test

The likelihood ratio test shows that the following independent variables (age of respondents [8.330; *p* = 0.016 *]; marital status—married [26.418; *p* < 0.001 ***]; household size 1 [24.617; *p* < 0.001 ***]; were significant at different levels. This implies that these independent variables contribute significantly to the overall model.

Regressed Parameters

*Mothers:*

With respect to the household food purchase decision making (dependent variable) and fathers as the reference variables, marital status “married” (for women or mothers) had a significant (b = −3.363, Wald = 7.975, *p* = 0.005 **) association with the household food purchase decision making (with a negative coefficient). This shows that women who are married among the study group were less likely to be the household food purchase decision maker. The odds ratio also indicated that women (mothers) were 0.035 times less likely to be the decision maker. Implying that amongst married couples within a household, women (mothers) are less likely to be the household food purchase decision maker. The following lists all other variables: influence of market choice (food price and food quality); age of respondent; challenges faced with food acquisition (traffic, time); occupation (fully employed); household size; educational status (Sec. school certificate and Bachelor degree) and household income per month after tax (1 and 3), had no significant effect on household food purchase decision making (with fathers as the reference variable) in the case of the mothers.

*Self (those who reside on their own):*

The variables regressed had no significant association with household food purchase decision maker with fathers as the reference variable. This conforms to expectation, since these groups of individuals live independently on their own and make their own food purchase decisions outside the influence of their parents (father or mother) or even the household in general terms. Supporting this finding is a study conducted in Kenya which found that the majority (52.7%) of households in their study were food insecure and households with male household heads were more likely to be food insecure than ones headed by their female counterparts [36]. In this Kenyan study, age, gender, and educational status (years of education) of the household head, land size, household size, and income significantly influenced household food insecurity so intra-household decision making had a vital role in influencing household food security. These findings indicate that gender-based resource allocation and women empowerment policies need to be enabled to improve household food security as women demonstrated better at household resource allocation than men [36]. In summary, this study shows that food insecurity persists in Awka, Anambra State. Respondents demonstrated a differentiation in primary factors for market choice and price, food quality and time availability had a mediating role. This suggests that culture and household preference and food choice also should be considered in the conceptualization of acquirability. These findings suggest that food insecurity is influenced by infrastructural and social factors which extend beyond availability, access and affordability; thus, the theme of acquirability is discussed next, in addition to how the framing of this concept may inform more focused policy interventions in the future.

## 5. Discussion

In reflecting on the extant literature and findings from this study, the proposed aspects or elements of acquirability that emerged were culture, household food preference and meal choice, time poverty, and resource availability and cooking skills.

### 5.1. Culture

Culture is the way of life of a given society. Culture is associated with the roles, positions and symbols of that society including societal beliefs, values, language, taboos, dress style, food and feeding habits, arts, language, and institutions [55]. Food is intimately associated with human culture and its influence on food security has remained a topic of discussion between researchers and policy makers in the fight against malnutrition, as food availability and access are associated with food norms, culture and practice within a certain environment [56]. Culture interreacts with food security via the different patterns or systems of crop production, utilization and distribution [57]. The household food decision maker is a key cultural factor identified in this study. In agreement, other studies [36,37] determined that men are more likely to make household food decision than women within a given household. Educational level of the household head and the number of relatives/extended family members were negatively associated with female perceptions of the food insecurity experienced by households [37]. Agada and Igbokwe’s study [55] showed that men (95%) had outright control over household income, with 78% of men also having preference over the sharing of household meal/food. Indeed, food culture and practices are strongly linked to household income [56]. Poor dietary diversity has also been linked to low-income urban households where the head of household was male [58].

### 5.2. Household Preference and Meal Choice

This factor considers the desire of individuals to consume a given type of food commodity. Household preferences will affect the household’s ability to acquire food as it influences food purchase decision at the household level. There is limited literature exploring the effect of food preference on food acquirability or food insecurity. However, inferences can be drawn from research with regard to consumers’ taste perceptions of specific food commodities. Research in Nigeria considered consumption of indigenous homegrown rice and found that consumers perceived indigenous rice as less attractive, suitable for poorer people compared to imported rice [59]. Indigenous rice was also seen to have specific quality problems such as contamination with sand, debris, stones, broken grains and an unpleasant odor. These perceptions can be difficult to overcome. Further research in Nigeria and Cameroon on plantain suggests that gender plays a role in perceptions of taste and food choice and nutrition was influenced by education and annual income [60]. Household size also played a key role in consumer choice, especially when price was also a consideration. The study suggested that there were a range of consumer segments for plantain differentiated by specific product requirements. Sensory appeal is thus a key acquirability factor affecting food choice in Nigeria [61,62,63].

### 5.3. Time Poverty

Time poverty implies that an individual perceives they do not sufficient discretionary time. Discretionary time enables restorative activities, supports personal capabilities and personal development [64], i.e., committed and necessary time [65], where committed time relates to time working, caring for children and others, the household and then the time necessary for wellbeing. Time poverty is often associated with convenience, especially with food consumers’ ability to afford food [5]. In this study, respondents from the supermarket setting cited time poverty as a key reason for purchasing food in that setting along with food quality, offsetting this against food price. Other research from Nigeria corroborates this finding, in that individuals who purchased food from supermarkets perceived pressures on time as a key driver for their choice of food location [66].

### 5.4. Resource Availability and Cooking Skills

This acquirability factor represents the differences in ability to cook, or possess cooking skills and cooking resources (energy, equipment, etc.), linking to food choice and the household’s ability to acquire food. Cooking refers to the activity that utilizes resources such as money, time, and other goods including utensils to prepare palatable and edible food [67]. Poor diet is linked to poor cooking skills [68]. The possession and application of cooking skills have several social benefits including financial saving on food budget, better diet quality, and ability to control weight [69,70]. Cooking also allows people make healthier food choices. While some studies have considered cooking practices in Nigeria, there is a lack of empirical data on how the lack of cooking skills influences food acquirability in urban locations. In this study, the sample population reported poor access to electricity and potable water as concerns. This will influence the ability to cook and the fuels used for cooking, given that the individuals have cooking skills associated with the resources available. Cooking skills influence how households acquire food. The consumption of vended street food in Nigeria was found to be associated with lack of cooking facilities [71].

In summary, the household decision maker, by virtue of income level, gender, age, employment and/or education, makes decisions on budgets for food purchase. This individual may not, however, make the best nutritional decisions for the household [36]. In the African context, the male household heads are typically involved with the budgeting for food purchase [36], but not necessarily its purchase or preparation, but where female heads of household are the food deciders, where men are absent, the household has a more balanced diet, and where intra-household food agency for women increases food insecurity is reduced [72]. This has implication for food acquirability.

Secondly, time poverty contributes to a household’s ability to acquire food [73]. Hence, some households may resort to the consumption of snacks, fast food and the patronage of roadside/food hawkers/street food vendors whose food is fast but not necessarily safe. Household food decision making may also be based on the culture and dietary preferences of household’ members [74]. These are important elements to consider when developing policies that would guide food decisions based on cultural influences. Palatability and taste attributes of food can importantly affect the acquirability of food by consumers [75]. The benefits of having the facilities to cook and also possess cooking skills is far reaching in terms of acquirability. The ability to cook encourages togetherness within a given household and supports the passing on of skills to other members of the household.

## 6. Conclusions

This paper considered the addition of a new pillar or element “food acquirability” to the existing pillars of food security (food availability, accessibility, utilization and stability). Food acquirability, as a proposed element of food security, considers factors that impact the ability to acquire food, despite it being physically and financially available and acceptable. While the focus on the production of food within national boundaries or via imports is important to ensure food availability, the availability of funds (income) to ensure access, utilization, and the stability of food within households is also crucial. However, the social factors explored in this paper can affect decisions at the household and intra-household level. This study seeks to inform further empirical research that could validate these elements of food acquirability, while proposing that policy makers consider food acquirability as one of the elements of food security in the future.

Food acquirability, as a new pillar of food security, has policy implications regarding the gendered exclusion of women from food purchasing decisions or the amount of money assigned at household level for purchasing food. Increased provision of electricity and potable water to support the urban cooking of food, as well as access to cooking, food preparation and storage facilities, would also be advantageous. More accessible information on cooking skills is important. Video programs could be aired to inform viewers about the various techniques and methods of preparing different dishes. Policies that favor the delivery of cooking classes in secondary schools across Africa could be encouraged. This is not to say that (some) countries in Africa are not currently doing this, but for other countries where such practice are yet to be put in place, this could be the game changer, as this action could equip young urban Africans with the required skills to have more agency over their food and nutritional security. Further empirical research should now explore the concept of food acquirability in the context of seeking to ensure food security at national, local and household level and the drivers of, and lived experience of, being food-insecure at household level. This work should focus both qualitatively and quantitatively on determining the factors that comprise food acquirability and how they can inform and improve future food policy.

## Figures and Tables

**Table 1:** Summary of variables from the study.

	Local Market		Supermarket		Combined Data	
Age Range	Frequency (n)	Percent (%)	Frequency (n)	Percent (%)	Frequency (n)	Percent (%)
18–25	8	13.3	10	16.7	18	15
26–45	45	75	42	70	87	72.5
46–65	7	11.7	8	13.3	15	12.5
Male	19	31.7	14	23.3	33	27.5
Female	41	68.3	46	76.7	87	72.5
Single	15	25	14	23.3	29	24.2
Married	38	63.3	42	70	80	66.7
Widowed	6	10	3	5	9	7.5
Divorced	0	0	1	1.7	1	0.8
Widower	1	1.7	0	0	1	0.8
**Decision maker**						
Father	33	55.0	34	56.7	67	55.8
Mother	12	20.0	15	25.0	27	22.5
Not residing in a family	15	25.0	11	18.3	26	21.7
**Household size**						
Household Size of 1	14	23.3	11	18.3	25	20.8
Household Size of 2	6	10	8	13.3	14	11.7
Household Size of 3–6 (Median 4.5)	27	45	32	53.3	59	49.2
Household Size of 7–10 (Median 8.5)	13	21.7	9	15	22	18.3
**Education**						
No formal education	6	10	0	0	6	5
Secondary school leaving Certificate	27	45	22	36.7	49	40.8
Bachelor’s Degree	18	30	26	43.3	44	36.7
Master’s Degree	3	5	5	8.3	8	6.7
Tertiary Institution—Polytech	6	10	7	11.7	13	10.8
**Employment**						
Unemployed	1	1.7	2	3.3	3	2.5
Partially Employed	6	10	2	3.3	8	6.7
Partly Employed and Student	9	15	8	13.3	17	14.2
Student	2	3.3	6	10	8	6.7
Self Employed	32	53.3	22	36.7	54	45
Fully Employed	10	16.7	20	33.3	30	25
**Household income per month after tax**						
<N18,000	7	11.7	10	16.7	17	14.2
N18,000–N40,000	23	38.3	15	25	38	31.7
N41,000–N80,000	20	33.3	18	30	38	31.7
N81,000–N100,000	6	10	14	23.3	20	16.7
N100,000>	4	6.7	3	5	7	5.8

**Table 2:** Factors of influence in food market choice/factors that impact the decision to purchase food.

	Local Market				Supermarket			
	Yes		No		Yes		No	
	Frequency (n)	Percentage (%)	Frequency (n)	Percentage (n)	Frequency (n)	Percentage (%)	Frequency (n)	Percentage (%)
Food price	55	91.7	5	8.3	4	6.7	56	93.3
Distance to market	26	43.3	34	56.7	17	28.3	43	71.7
Food Variety	21	35	39	65	7	11.7	53	88.3
Loyalty	10	16.7	50	83.3	10	16.7	50	83.3
Food Quality	5	8.3	55	91.7	58	96.7	2	3.3

**Table 3:** Respondents’ reflection on challenges with acquiring food.

	Local Market (Eke Awka)		Supermarket		Combined Data Set	
	Frequency (n)	Percent (%)	Frequency (n)	Percent (%)	Frequency (n)	Percent (%)
Road Traffic	30	50	14	23.3	44	36.7
Bad road network	19	31.7	7	11.7	26	21.7
Time	8	13.3	32	53.3	40	33.3
Distance to Market	2	3.3	1	1.7	3	2.5
High transportation cost	1	1.7	4	6.7	5	4.2
No Challenge Faced	0	0	2	3.3	2	1.7
Total	60	100	60	100	120	100.0

**Table 4:** Descriptive statistics for variables regressed (food consumers).

	N	Minimum	Maximum	Mean	Std. Deviation
Household Food Purchase Decision	120	1	3	1.66	0.815
Influence of Market Choice (Food Price)	120	0	1	0.49	0.502
Influence of Market Choice (Quality of Food)	120	0	1	0.53	0.501
Age of Respondent	120	1	3	1.98	0.526
Marital Status: Married	120	0	1	0.67	0.473
Challenges: Traffic	120	0	1	0.41	0.494
Challenges: Time	120	0	1	0.35	0.479
Fully Employed	120	0	1	0.46	0.500
Household Size 1	120	0	1	0.33	0.470
ED- Sec. School Certificate	120	0	1	0.46	0.500
ED- Bachelor Degree	120	0	1	0.37	0.484
Household Income PMAT 1	120	0	1	0.46	0.500
Household Income PMAT 3	120	0	1	0.23	0.419
Frequency of Market Visit	120	1	3	1.87	0.607
Valid N (listwise)	120				

PMAT= Per month after tax; ED = Educational degree.

**Table 5:** Regression coefficient: considering household food decision maker-marital status (married).

Household Food Purchase Decision Maker		B	Wald	Sig.	Exp(B)
Mother	Intercept	10.481	5.622	0.018 *	
	Influence of Market Choice (Food Price)	−0.927	0.537	0.464	0.396
	Influence of Market Choice (Quality of Food)	0.745	0.272	0.602	2.106
	Age of Respondent	−2.661	3.413	0.065	0.070
	Marital Status: Married	−3.363	7.975	0.005 **	0.035
	Challenges in acquiring Food: Traffic	0.135	0.028	0.866	1.144
	Challenges in Acquiring Food: Time	−1.083	1.360	0.244	0.338
	Fully Employed	−0.333	0.211	0.646	0.717
	Household Size 1	−0.541	0.312	0.576	0.582
	ED- Sec. School Certificate	−0.781	0.731	0.393	0.458
	ED- Bachelor Degree	−1.111	1.537	0.215	0.329
	Household Income PMAT 1	0.180	0.062	0.804	1.197
	Household Income PMAT 3	−1.302	1.807	0.179	0.272
	Frequency of Market Visit	−0.874	1.306	0.253	0.417
Self	Intercept	14.061	0.000	1.000	
	Influence of Market Choice (Food Price)	33.193	0.000	0.998	2603.000
	Influence of Market Choice (Quality of Food)	17.494	0.000	0.997	3958.216
	Age of Respondent	−39.011	0.000	0.997	1.142
	Marital Status: Married	−52.209	0.000	0.995	2.118
	Challenges in acquiring Food: Traffic	−36.053	0.000	0.996	2.198
	Challenges in Acquiring Food: Time	−4.168			0.015
	Fully Employed	−50.160	0.000	0.994	1.644
	Household Size 1	66.841	0.000	0.994	1.068
	ED- Sec. School Certificate	51.020	0.000	0.995	1.437
	ED- Bachelor Degree	36.413			6514.000
	Household Income PMAT 1	33.185	0.000	0.995	2583.000
	Household Income PMAT 3	30.977			2839.400
	Frequency of Market Visit	−17.582	0.000	0.997	2.312
a. The reference category is Father.					

* Significant at *p* < 0.05, ** Significant at *p* < 0.01%, Adjusted R^2^ = 0.695 F-value = 42.836.

## Data Availability

The original contributions presented in the study are included in the article, further inquiries can be directed to the corresponding author.
